# Comprehensive Genomic Characterization of Tumor Microenvironment and Relevant Signature in Clear Cell Renal Cell Carcinoma

**DOI:** 10.3389/fonc.2022.749119

**Published:** 2022-05-16

**Authors:** Chuanjie Zhang, Feng Qi, Yuxiao Zheng, Xin Xia, Xiao Li, Xinwei Wang

**Affiliations:** ^1^ Department of Urology, Ruijin Hospital, Shanghai Jiao Tong University School of Medicine, Shanghai, China; ^2^ Department of Urology, Jiangsu Cancer Hospital and Jiangsu Institute of Cancer Research and Affiliated Cancer Hospital of Nanjing Medical University, Nanjing, China; ^3^ Department of Anatomy, Nanjing Medical University, Nanjing, China; ^4^ Department of Medical Oncology, Jiangsu Cancer Hospital and Jiangsu Institute of Cancer Research and Affiliated Cancer Hospital of Nanjing Medical University, Nanjing, China

**Keywords:** ccRCC, TMEscore, TCGA, ICGC, immunotherapy

## Abstract

**Purpose:**

To systematically investigate the characterization of tumor microenvironment (TME) in clear cell renal cell carcinoma (ccRCC), we performed a comprehensive analysis incorporating genomic alterations, cellular interactions, infiltrating immune cells, and risk signature.

**Patients and Methods:**

Multi-omics data including RNA-seq, single-nucleotide variant (SNV) data, copy number variation (CNV) data, miRNA, and corresponding prognostic data were obtained from The Cancer Genome Atlas (TCGA) and the International Cancer Genome Consortium (ICGC) database. The CIBERSORT algorithm was utilized to identify prognostic TME subclusters, and TMEscore was further quantified. Moreover, the mutational landscape of TCGA-KIRC was explored. Lastly, TIDE resource was applied to assess the significance of TMEscore in predicting immunotherapeutic benefits.

**Results:**

We analyzed the TME infiltration patterns from 621 ccRCC patients and identified 5 specific TME subclusters associated with clinical outcomes. Then, we found that TMEcluster5 was significantly related to favorable prognosis and enriched memory B-cell infiltration. Accordingly, we depicted the clustering landscape of TMEclusters, TMEscore levels, tumor mutation burden (TMB), tumor grades, purity, and ploidy in all patients. Lastly, TIDE was used to assess the efficiency of immune checkpoint blockers (ICBs) and found that the TMEscore has superior predictive significance to TMB, making it an essential independent prognostic biomarker and drug indicator for clinical use.

**Conclusions:**

Our study depicted the clustering landscape of TMEclusters, TMEscore levels, TMB, tumor grades, purity, and ploidy in total ccRCC patients. The TMEscore was proved to have promising significance for predicting prognosis and ICB responses, in accordance with the goal of developing rationally individualized therapeutic interventions.

## Introduction

Kidney cancer is a common urological malignancy worldwide, in which clear cell renal cell carcinoma (ccRCC) accounts for nearly 85% of all diagnosed cases, along with increasingly annual cancer-related deaths (1). Currently, surgical intervention, incorporating laparoscopic partial nephrectomy (LPN) or radical nephrectomy (RN), remains the treatment of choice (2, 3). Moreover, increasing studies have brought intensive insights into the immune-related therapeutic targets, especially the significant role of tumor microenvironment (TME) in cancer progression and drug responses (4–6). The structure of TME mainly consisted of parental tumor cells, tumor-associated macrophages (TAMs), mesenchymal stem cells (MSCs), dendritic cells (DCs), cytotoxic T cells, helper T cells, and other inflammatory-related factors, which were robustly demonstrated to be associated with prognosis across multiple malignancies, including urothelial cancer, lung cancer, gastric cancer, melanoma, and breast cancer (7–9). Meanwhile, TME heterogeneity was a pivotal determinant for therapeutic efficiency and exhibited different profiles during the process of tumor progression (10, 11).

ccRCC was significantly correlated with immune infiltration and precious intensive efforts were devoted to explore more sensitive biomarkers to improve immunotherapy precision, including immune-related signature, tumor mutation burden (TMB), and other specific immune cells (12–14). To date, we could utilize the advanced computational methods to infer the abundance of immune cells and other cell types, including ssGSEA, CIBERSORT, and ESTIMATE (15–17). Several cohorts investigated the clinical efficiency of TME, and potential mechanisms uncovering the TME and immunotherapy response have been well experimentally characterized across several malignancies (18). Job et al. explored the heterogeneity of tumor stromal composition and developed a TME-based classification of intrahepatic cholangiocarcinoma to detect potentially targetable cancer subtypes (19). Dai et al. identified extracellular KRAS^G12D^ that acted as a hub mediator of cancer cell–macrophage communication and also provided a novel KRAS-targeted anticancer strategy based on TME (20). Furthermore, Wu et al. further demonstrated that gemcitabine treatment resulted in metabolic changes in residual tumor cells, leading to the resistance to T-cell-mediated killing (21). However, the comprehensive landscape of TME in ccRCC and the potential relationships of TME with genomic mutation burden or other related risk signatures have been limitedly elucidated. In addition, Programmed death ligand 1 (PD-L1, CD274), expressed on tumor and/or immune cells in the TME, interacts with PD-1 on tumor-infiltrating lymphocytes, attenuating effector T-cell responses and allowing tumors to escape immune attack (22, 23). Previous studies have indicated that aberrant expressions of TME-related genes could trigger TME remodeling, thereby impacting immune checkpoint blocker (ICB) efficacy. For instance, Siglec-15 suppresses antigen-specific T-cell responses *in vitro* and *in vivo*. Targeting Siglec-15 amplifies anti-tumor immunity in the TME, functioning as a potential target for normalization of cancer immunotherapy (24). Based on the *in vivo* epigenetic *CRISPR* screen, Li et al. identified Asf1a as a key modulator of lung adenocarcinoma sensitivity to anti-PD-1 therapy. Asf1a deficiency synergized with anti-PD-1 immunotherapy by promoting M1-like macrophage polarization and T-cell activation (25). Therefore, whether TME-related signature could predict ICB efficacy of RCC is a promising and interesting project to deal with.

In the present research, we adopted two well-characterized algorithms to infer the specific fractions of 22 immune cells based on the renal cell carcinoma cohort gene expression profiles (26, 27). The TME infiltration patterns of 683 tumors extracted from patients were well estimated and we systematically correlated the TME sub-clusters with genomic characteristics, somatic mutation profiles, clinical or pathological features of ccRCC, and drug responses. Accordingly, we further conducted a methodology to quantify the TME infiltration pattern as the TMEscore, which was assessed from multiple aspects to be a robust prognostic marker or predictive factor for response to immune-checkpoint inhibitors in ccRCC.

## Materials and Methods

### Kidney Cancer Datasets and Data Preprocessing

We carefully screened the kidney cancer databases and filtered out the samples with incomplete genomic or clinical information. In total, we obtained the KIRC RNA-seq (*N* = 530), single-nucleotide variant (SNV) data (*N* = 417), copy number variation (CNV) data (*N* = 534), miRNA (*N* = 592), and corresponding prognostic data (screened only with survival information, *N* = 527) from The Cancer Genome Atlas (TCGA) database (https://portal.gdc.cancer.gov/projects/TCGA-KIRC). Moreover, we downloaded the RNA-seq of 91 samples with clinical data from the International Cancer Genome Consortium (ICGC) database (https://dcc.icgc.org/projects/RECA-EU). In addition, the Fragments Per Kilobase Million (FPKM) format of RNA-seq data from TCGA-KIRC was transformed into TPM (Transcripts Per Million), and the Reads Per Kilobase Million (RPKM) format of RNA-seq data from ICGC was normalized into TPM. Lastly, the patients with incomplete survival information or expression data were excluded in this study.

### Estimation of Infiltrating Immune Cells in TME and Consensus Clustering

CIBERSORT (https://cibersort.stanford.edu/) is a developed algorithm utilized for deconvolving the expression matrix of immune cell subtypes based on the principle of linear support vector regression. Therefore, we pre-processed the RNA-seq (TPM normalized) data to infer the proportion of immune cells in tumor samples using the CIBERSORT algorithm and the LM22 gene signature. According to the proportion of immune cells analyzed by the CIBERSORT tool, we only selected the samples with *p* < 0.05 (*N* = 512 from total 618 samples). Based on the elbow (the square error of the WSSE group, which was obtained by looking for the “elbow point”) and gap statistics (the point where *Wk* falls the fastest, the value of *k* corresponding to the maximum value of Gap), we used the ConsensusClusterPlus package to find the TMEcluster with the best optimized *K* categories. Moreover, we conducted a total of 1,000 times iterations to achieve the robustness and stability of results and evaluate the potential relativity between survival outcomes and the classifications.

According to the TMEcluster results, the clustering results were mapped to RNA-seq data, and the limma package was utilized to search differentially expressed genes (DEGs) with *p* < 1e-03 and |log_2_FC|>2 as the cutoff value. We thus chose the class-specific differential genes and obtained the cluster signature genes using the random forest method to remove the redundant genes. Functional enrichment analysis was performed to observe the significant pathways that these signatures might be involved in. We referred to the algorithm introduced by Sotiriou et al. (28) and Cox regression model to calculate the TMEscore in ccRCC as the following: TMEscore = Ʃ log_2_(X+1) - Ʃ log_2_(Y+1), where X is the expression value of the positive gene set of Cox coefficient and Y is the expression value of the gene set of Cox coefficient. Last, we sought the best breakpoint of TMEscore using the maxstat package and then divided the samples into TMEscore-high and TMEscore-low to further analyze the prognosis between two main types.

### The Mutational Landscape of TCGA-KIRC

According to the downloaded TCGA-KIRC single-nucleotide polymorphism (SNP) data (*N* = 336 with TCGAbiolinks package), CNV data (*N* = 534), methylation data (*N* = 483), and survival data (*N* = 539 with cgdsr package), we intersected them with the RNA-seq data and finally derived 210 samples.

A variety of mutation types existed in cancers, including C > A, C > G, C > T, T > A, T > C, and T > G. Based on the six basic mutational types, we then considered one base upstream and downstream of the mutation site. Each base had four categories of A, T, C, and G; thus, a total of 96 mutation types were obtained with 4 * 6 * 4. In order to investigate different types of mutations, researchers introduced the concept of “Mutational Signature” and have already identified a total of 21 various Mutational Signatures across 30 malignancies. The frequencies of the 96 mutational types in different cancers were different. Combined analysis of the occurrence of 96 mutation types can be used as a fixed mutation pattern. Several Mutational Signatures were currently included in the COSMIC database, some of which were known, such as signature4 and signature29, and were related to exposure to the smoking environment. Accordingly, we used the maftools package (https://bioconductor.org/packages/release/bioc/html/maftools.html) and the SomaticSignatures package (https://bioconductor.org/packages/release/bioc/html/SomaticSignatures.html) to map the mutational landscape and characteristics in ccRCC.

The GISTIC algorithm was used to detect common CNV regions in all samples, including chromosome arm horizontal CNV and the smallest common region across samples. The GISTIC algorithm parameter determined the peak interval with the 0.95 as the confidence level, and the others remained default parameters. We selected the corresponding GISTIC module in GenePattern to conduct the analysis. Meanwhile, we utilized the ABSOLUTE package (https://software.broadinstitute.org/cancer/cga/absolute_download) to conduct the tumor purity and ploidy analysis based on the CNV results. The ABSOLUTE algorithm was applied to calculate scores from the pre-designed cancer karyotype and somatic mutation frequency and then integrated them, from which the highest score was the optimal model. Then, the tumor purity and ploidy were accordingly inferred.

### Integrative Analysis in TME of ccRCC

**Figure 1:**
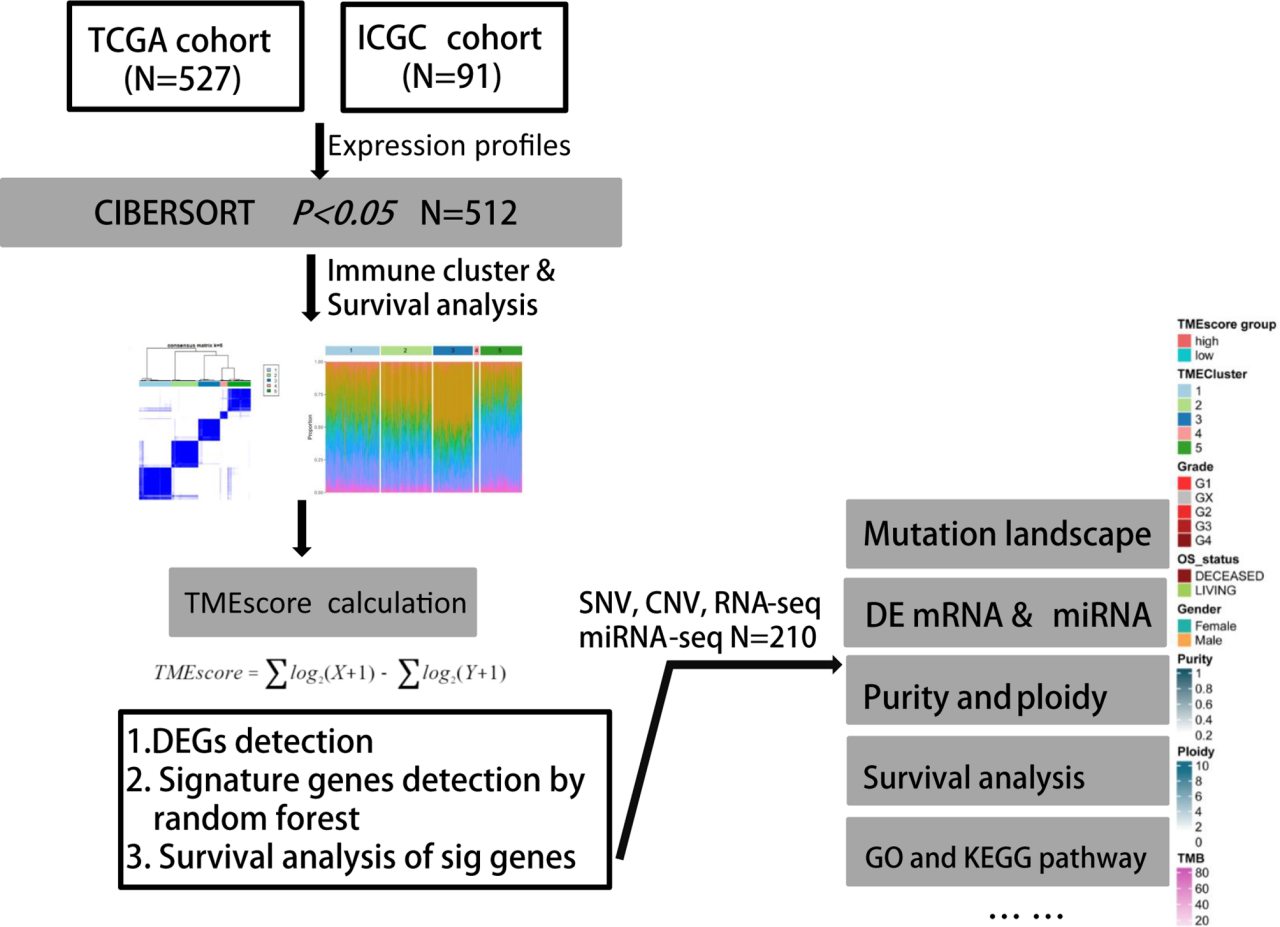
Flowchart of analytical process of TME phenotypes and TMEscore in our study based on two independent ccRCC cohorts.

Firstly, we identified the differential transcriptome of miRNA or mRNA across several clusters and conducted functional enrichment analysis to uncover the specific biological processes in different TME subgroups. Furthermore, we conducted prognostic analysis of differential genes in the TME-high- and TME-low groups. We aimed to depict the molecular and clinical characteristics across TME subgroups from comprehensive omics data. Finally, the TIDE tool (http://tide.dfci.harvard.edu/) from Harvard University was used to assess the clinical efficiency of immune checkpoint inhibition therapy, where higher TIDE predictive scores correlated with poor therapeutic effect and worse prognosis. The whole process of analysis was illustrated in **Figure 1**.

## Results

### Landscape of Infiltrating Immune Cells in TME and Clustering Analysis

**Figure 2:**
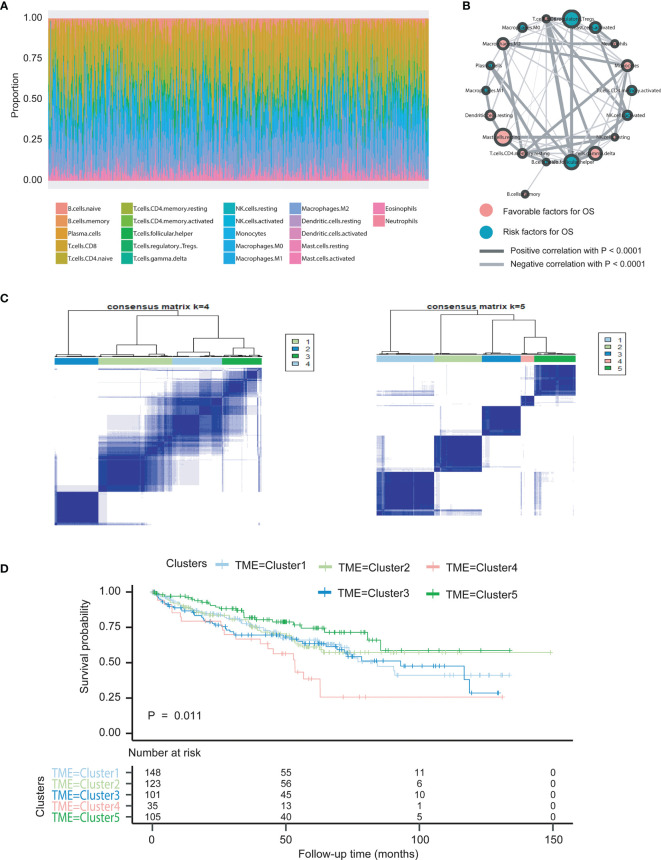
Illustration of landscape of TME in ccRCC patients and characterization of TME subclusters. **(A)** The specific 22 immune fractions represented by different colors in each sample were shown in barplot via CIBERSORT algorithm. **(B)** Cellular interaction of cell types in tumor micro environment. Favorable factors for overall survival were indicated in pink, and hazard factors for overall survival were shown in dark green. The lines connecting TME cells represented their cellular interactions, where the thickness of the lines indicated the strength of correlation calculated by Spearman correlation analysis. Positive correlation was indicated in black and negative correlation in silver. **(C)** Clustering analysis based on ConsensusClusterPlus package was conducted and indicates the optimized categories were 5 for classifications of TME phenotypes. **(D)** Kaplan-Meier curves with log-rank test for overall survival (OS) of all cancer patients from seven two cohorts (TCGA, ICGC) with the TME infiltration classes. The number of patients in TMEcluster 1, 2, 3, 4 and 5 phenotypes were n=148, n=123, n=101, n=35, and n=105, respectively. Log-rank test showed an overall statistical result of P=0.011, where patients with TMEcluster 5 were with the most favorable outcomes.

**Figure 3:**
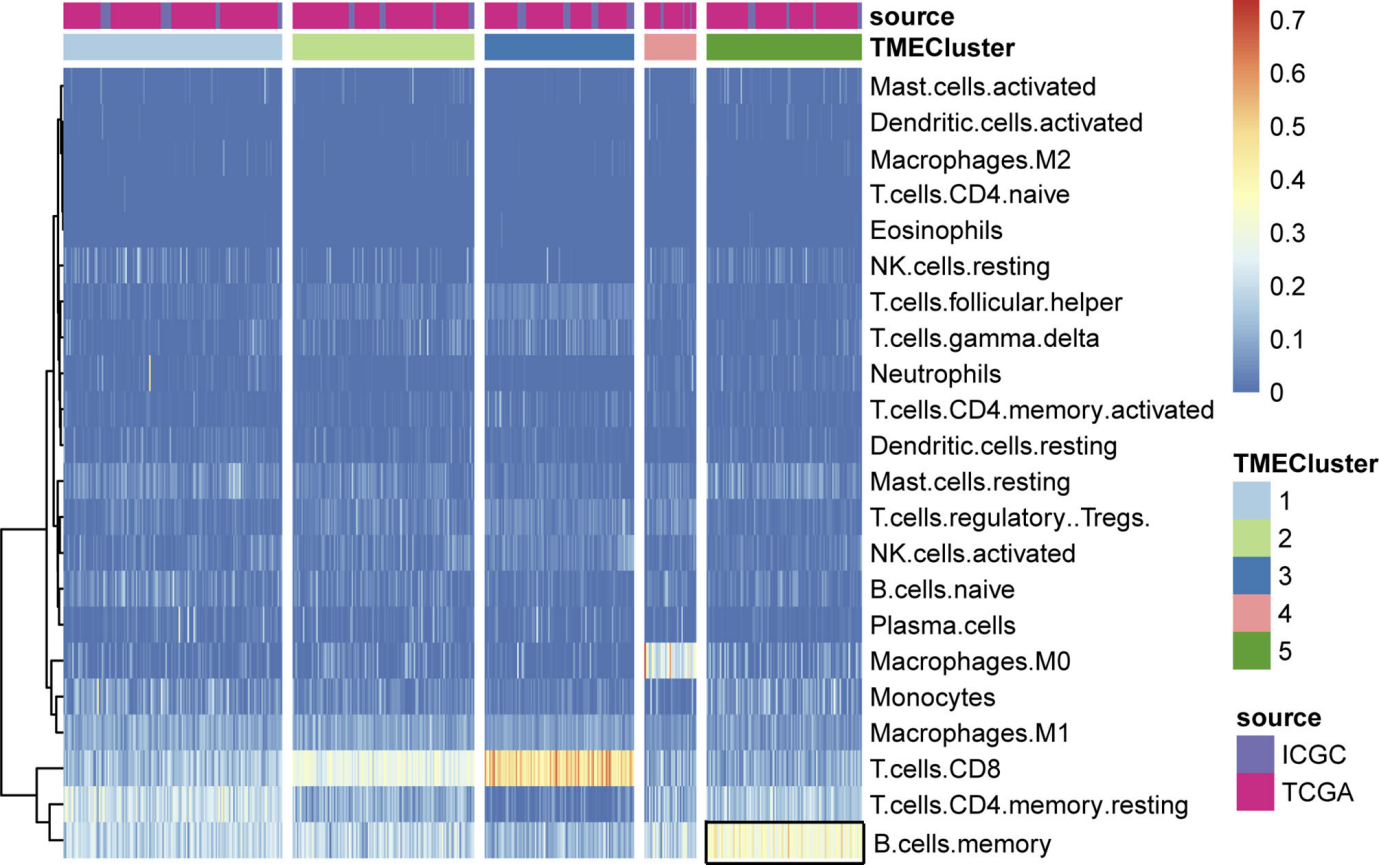
Unsupervised clustering of TME cells for patients in the two cohorts. We assessed the differential infiltrating levels of immune cells in various clusters, where the memory B cells exhibited significantly enrichment in TME cluster5.

According to the integrated RNA-seq data (*N* = 618), we utilized the CIBERSORT package within 1,000 interactions to obtain the differential fractions of various immune cells across 512 samples, including CD4 cell, CD8 cell, B cell, and monocytes (**Figure 2A**). The clinical characteristics of all 618 ccRCC patients included in this study were shown in **Table 1**. We further depicted the potential associations among immune cells and their respective prognostic significance. We found that the Tregs cells and T follicular helper cells correlated negatively with survival outcomes, while Mast resting cells and T gamma delta cells were associated positively with prognosis in Cox regression models (*p* < 0.0001, **Figure 2B**; **Table S1**). Moreover, we combined with ConsensusClusterPlus package and iterated 1,000 times (*K* = 1:10) to stabilize the classification categories, obtaining the optimized classification of the samples (**Figure S1**). We observed that the TMEcluster classifications were significantly associated with survival outcomes when *k* = 5, among which the TMEcluster4 (*N* = 35) had the worst prognosis and TMEcluster5 (*N* = 105) had the best prognosis (**Figure 2C**). We further compared the immune cell proportions across different TMEclusters and found the significant survival differences across these 5 clusters (**Figures 2D**, **3**; **Figure S2**).

**Table 1:** Clinical characteristics of all 618 ccRCC patients included in this study.

Variables	TCGA	ICGC
	(*N* = 537)	(*N* = 91)
**Age (mean ± SD)**	60.59 ± 12.14	60.47 ± 9.97
**Follow-up (years)**	3.12 ± 2.23	4.14 ± 1.73
**Status**		
Alive	367 (68.34)	61 (67.03)
Dead	170 (31.66)	30 (32.97)
**Gender**		
Male	346 (64.43)	52 (57.14)
Female	191 (35.57)	39 (42.86)
**AJCC-T**		
T1	275 (51.21)	54 (59.34)
T2	69 (12.85)	13 (14.28)
T3	182 (33.89)	22 (24.18)
T4	11 (2.05)	2 (2.20)
**AJCC-N**		
N0	240 (44.69)	79 (86.81)
N1	17 (3.17)	2 (2.20)
Unknow	280 (52.14)	10 (10.99)
**AJCC-M**		
M0	426 (79.33)	81 (89.01)
M1	79 (14.71)	9 (9.89)
Unknow	32 (5.96)	1 (1.10)
**Pathological stage**		
I	269 (50.09)	–
II	57 (10.61)	–
III	125 (23.28)	–
IV	83 (15.46)	–
Unknow	3 (0.56)	–
**Grade**		
G1	14 (2.61)	–
G2	230 (42.83)	–
G3	207 (38.54)	–
G4	78 (14.53)	–
Unknow	8 (1.49)	–
**TMB levels**		
Low	109 (20.30)	–
High	101 (18.81)	–
Unknown	327 (60.89)	

Data are shown as n (%).

TCGA, The Cancer Genome Atlas; ICGC, International Cancer Genome Consortium; AJCC, American Joint Committee on Cancer.

### Identification of Significant Prognostic Signature Associated With TME

**Figure 4:**
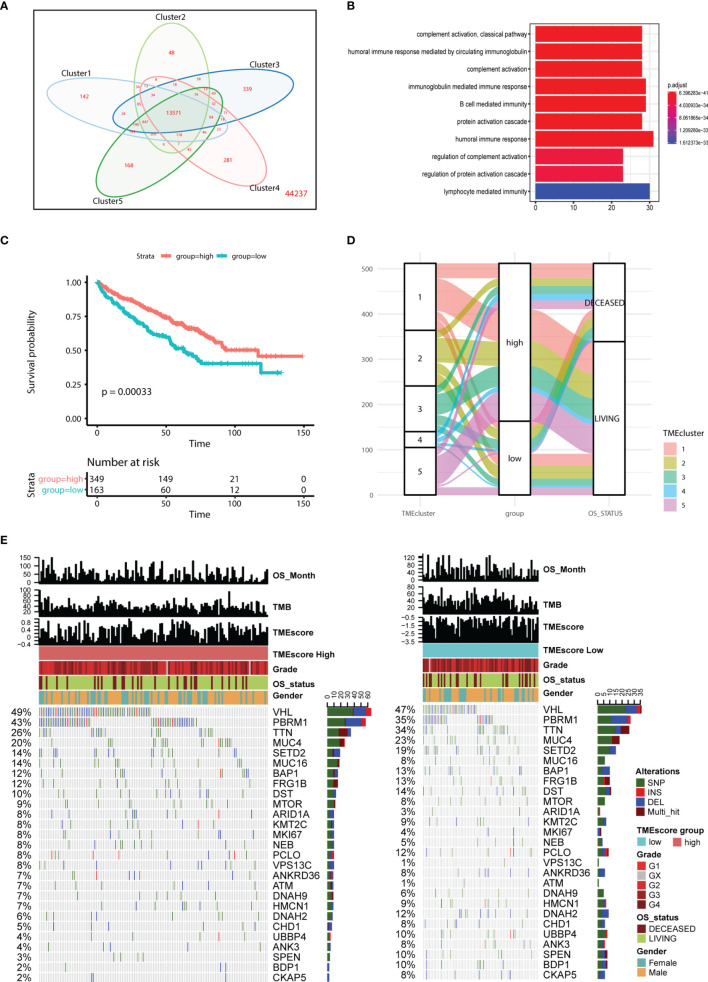
Construction of TMEscore and integrative analysis in TME. **(A, B)** Venn diagram showed the totally 13571 intersected genes, in which functional enrichment analysis indicates the genes might be involved in B cell mediated immunity, humoral immune response, lymphocyte medicated immunity and other cancer-related immune crosstalk. **(C, D)** Kaplan-Meier analysis and mulberry map indicate the patients in low TMEscore group correlate with poor survival outcomes (log-rank test, P < 0.001). **(E)** Mutated profiles of top variant genes correlated with TMEscore. Single nucleotide variants: dark green, InDel (insertion or deletion): red, frameshift: blue, Multi_hit: brown. TMEscores, TCGA molecular subtypes, TMEscore, gender, and overall survival status are shown as specific annotations.

We set the cutoff value with *p* < 1e-3 and |logFC| > 2 to screen the DEGs and obtained a total of 13,571 genes across different clusters (**Figure 4A**; **Table S2**). Functional analysis indicated that these genes were mainly involved in humoral immune response, immunoglobulin-mediated immune response, and B-cell-mediated immunity (**Figure 4B**). Additionally, we utilized the Cox regression model to analyze the genes with survival outcomes and divided the samples into two groups, namely, TME-high and TME-low groups. Kaplan–Meier analysis indicated that patients in the TME-low group suffered from worse prognosis than those in the TME-high group (**Figure 4C**). The Sankey diagram also revealed the potential associations among TMEcluster, groups, and vital status (**Figure 4D**; **Table S3**).

### Profiles of Cancer Somatic Genome and Relationships With TME

**Figure 5:**
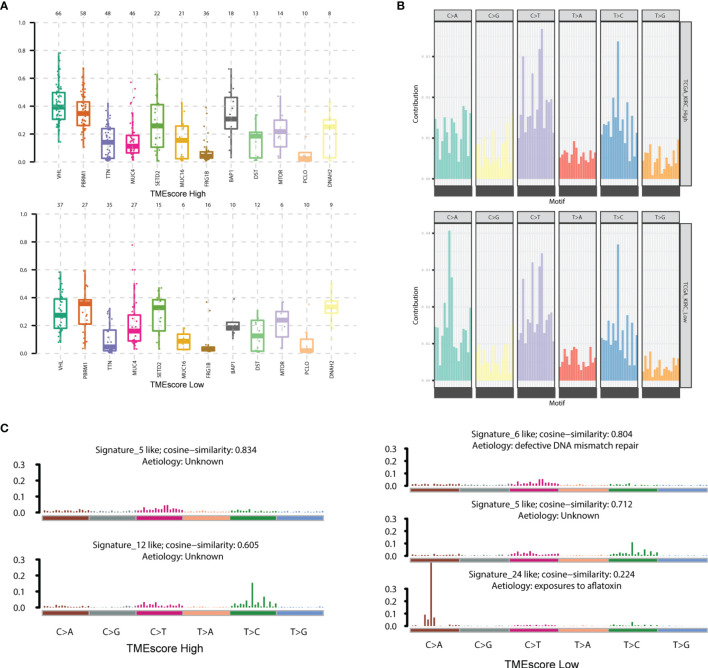
Characterization of mutated profiles and related signature between two TMEscore groups. **(A)** The oncogenetic drivers of UBBP4, SPEN and BDP1 exhibited significantly differential mutated frequencies between two groups and we illustrated the results in boxplot. **(B)** We accordingly classified the mutations into 96 types and calculated the distributed frequencies in 210 KIRC samples. **(C)** We extracted the features and compared them with included signature in COSMIC dataset. The mutated features in TME-high group were mainly associated with signature5, signature12, while those in TME-low group correlated with signature5, signature6, signature24. The signature6 was found to be involved in DNA mismatch repair defect, and signature 24 was associated with Aflatoxin exposure.

We performed the statistical analysis of the mutation data in 210 tumor samples, including mutation annotations, proportions of base change, and the top 10 drivers (**Figure S3**). In KIRC, the missense mutations occurred the most frequently, and the main types were SNP, followed by DEL and INS (**Figure S3**). In these samples, the top 10 mutated drivers included VHL, TTN, and PBRM1 (**Figure S3**). We further divided the patients into TME-high and TME-low groups, and the differential mutated profiles are illustrated in **Figure 4E**. Accordingly, we observed that VHL harbored the most mutated sites in KIRC distributed across nearly half of the cases. Furthermore, the oncogenetic drivers of UBBP4, SPEN, and BDP1 exhibited significantly differential mutated frequencies between two groups, and we illustrated the results in boxplots (**Figure 5A**). We then classified the mutations into 96 types and calculated the distributed frequencies in 210 KIRC samples (**Figure 5B**; **Table S4**). The relationships between mutated frequencies and specific signature were estimated based on the COSMIC dataset. We performed the non-negative matrix factorization incorporating the 210 samples as the rows and the 96 mutated types as the columns. We extracted the features and compared them with included signature in COSMIC. The mutated features in the TME-high group were mainly associated with signature5 and signature12, while those in the TME-low group correlated with signature5, signature6, and signature24. Signature6 was found to be involved in DNA mismatch repair defect, and signature 24 was associated with aflatoxin exposure (**Figure 5C**).

**Figure 6:**
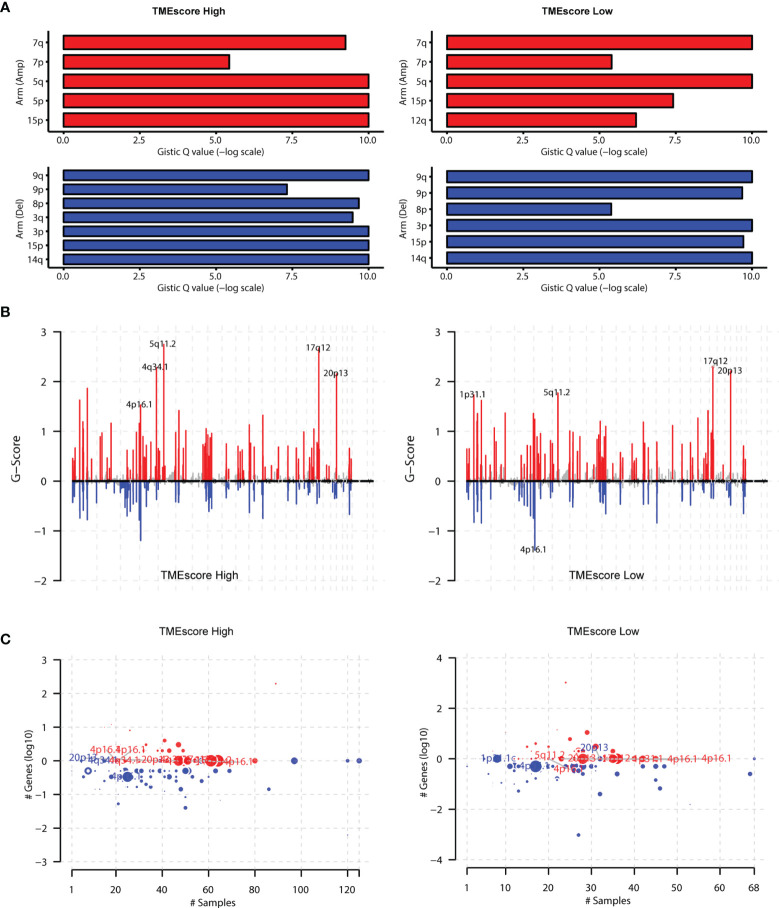
Assessment of copy number variation (CNV) burden in two TMEscore groups. **(A)** We also conducted the GISTIC software to analyse the copy number variations in two groups and found that the amplification of 15p, 5q, 5p and deletions of 9q, 15p, 14q were the most prominent in TME-high group. However, the amplification of 7q, 5q and the deletions of 9q, 3p, 14q were the most significant in the TME-low group. **(B)** Besides, we totally detected 102 amplifications TME-high samples (MCRs, q-value<1e-4), among which 5q11.2, 17q12, and 4q34.1 were the most significantly amplified regions. In TME-low samples, we totally detected 87 amplifications and found 17q12, 20p13, 5q11.2 were the most significantly amplified regions. **(C)** The 79 copy number deletions in TME-high samples were detected, among which 4p16.1, 3q29, and 1q21.3 were the most significantly deleted regions. However, 1q21.3, 12p13.31 were the most significantly deleted regions in TMEscore low group.

We also used the GISTIC software to analyze the CNV in two groups and found that the amplification of 15p, 5q, and 5p and the deletions of 9q, 15p, and 14q were the most prominent in the TME-high group. However, the amplification of 7q and 5q and the deletions of 9q, 3p, and 14q were the most significant in the TME-low group (**Figure 6A**). Moreover, we totally detected 102 amplifications and 79 copy number deletions in TME-high samples (MCRs, *q*-value < 1e-4) in **Figure 6B**. Among them, 5q11.2, 17q12, and 4q34.1 were the most significantly amplified regions and 4p16.1, 3q29, and 1q21.3 were the most significantly deleted regions. In TME-low samples, we totally detected 87 amplifications and 58 copy number deletions, among which 17q12, 20p13, and 5q11.2 were the most significantly amplified regions and 4p16.1, 1q21.3, and 12p13.31 were the most significantly deleted regions (**Figure 6C**). The tumor purity and ploidy of the samples were estimated based on the ABSOLUTE software. The range of tumor purity was 0.28–1 and the ploidy of the tumor cell genome was 1.78–9.06, indicating that the genome disorders occurred commonly during tumorigenesis (**Table S5**). Furthermore, Wilcoxon test suggested that no significant difference was observed between high and low TMEscore groups in **Figure S4**.

### Comprehensive Results and Significance of TMEscore in Predicting Immunotherapeutic Benefits

**Figure 7:**
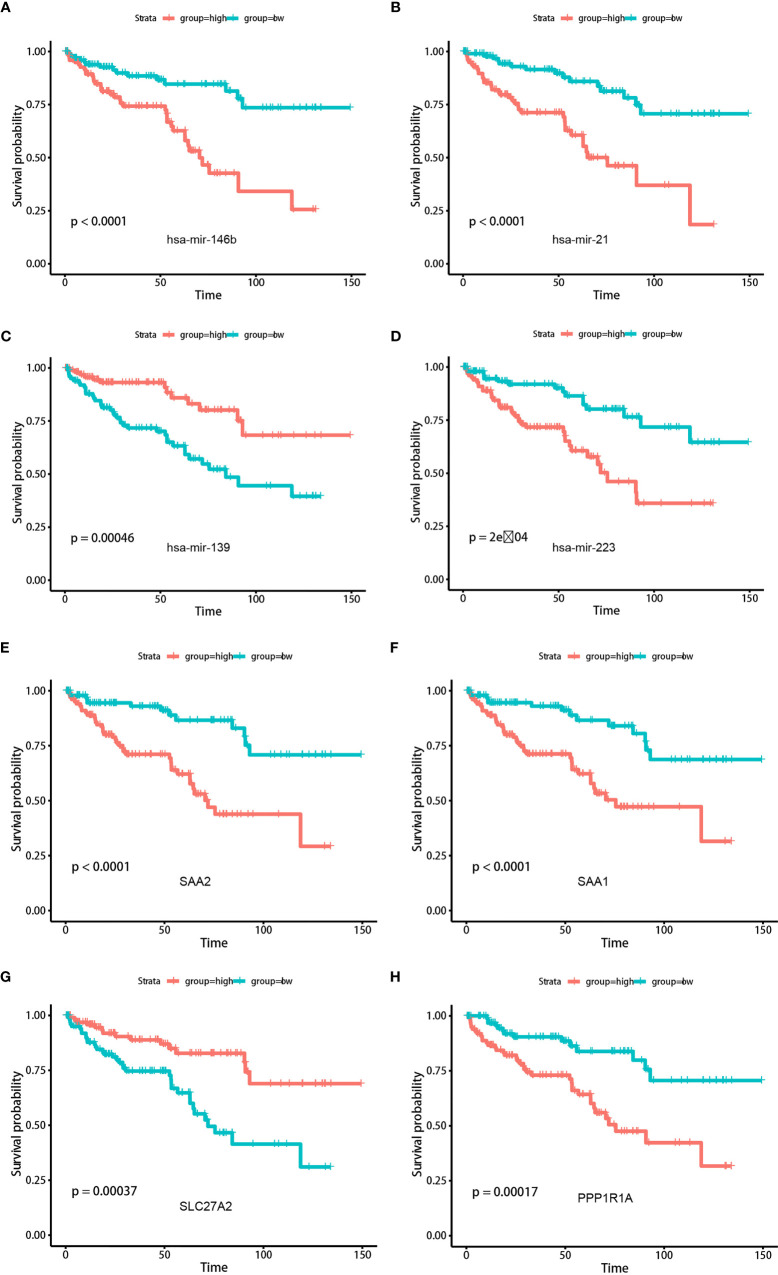
Kaplan-Meier analysis was conducted to assess the significantly risk signature expressed differentially (top miRNAs and mRNAs) in two TMEscore groups. **(A)** hsa-mir-146b. **(B)** hsa-mir-21. **(C)** hsa-mir-139. **(D)** hsa-mir-223. **(E)** SAA2. **(F)** SAA1. **(G)** SLC27A2. **(H)** PPP1R1A.

**Figure 8:**
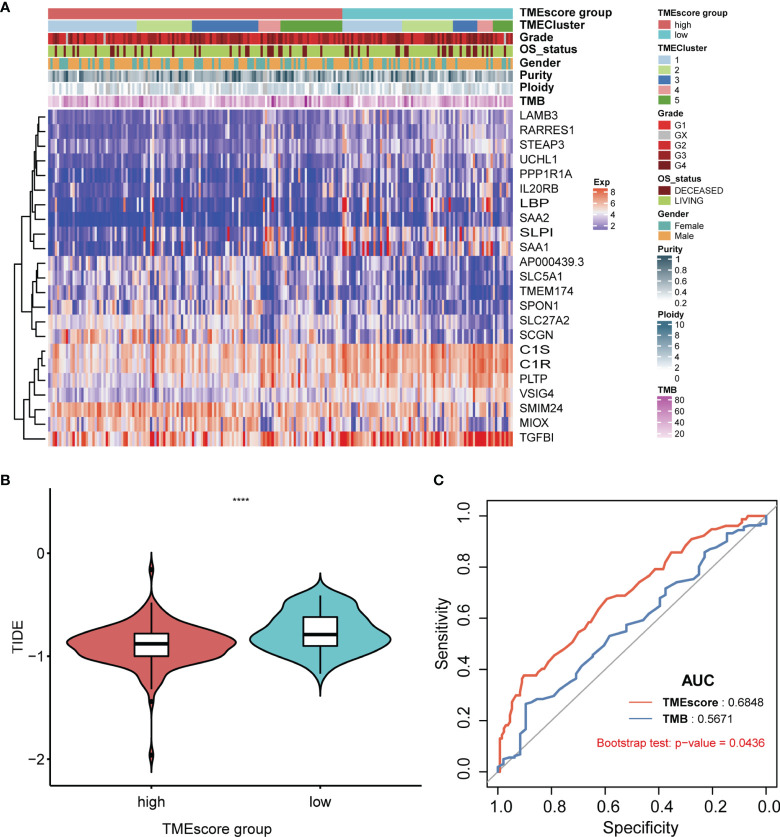
Systematic exhibition of TMEscore with other variables and associations with ICB efficiency compared with TMB. **(A)** Illustration of comprehensive genomic landscape integrating TMEcluster, TMEscores, mutation features (purity, ploidy, TMB) and clinical characteristics (age, gender, overall status). **(B)** In the current analysis, we found that KIRC patients with higher TME-scores might benefit from better ICBs efficiency and optimistic prognosis. **(C)** Compared with traditional indicator of TMB, TMEscore possess the more predictive sensitivity based on the ROC curve with 0.684.

Differential analysis revealed a total of 45 differentially expressed miRNAs and 163 mRNAs between TME-high and TME-low groups (**Tables S6**, **S7**). Functional enrichment analysis suggested that these differential genes were mainly involved in B-cell-mediated immunity, humoral immune response, and other cancer-related pathways (**Figures S5**, **S6**; **Table S8**). The log-rank test revealed 19 survival-related miRNAs and 23 mRNAs, including hsa-mir-21, hsa-mir-223, hsa-mir-146b, hsa-mir-139, SAA1, SLC27A2, PPP1R1A, and SAA2 (**Figure 7**; **Table S9**). We further illustrated the comprehensive genomic landscape integrating TMEcluster, TMEscores, mutation features (purity, ploidy, and TMB), and clinical characteristics (age, gender, and overall status) in **Figure 8A** and **Table S10**. The TIDE dataset was utilized to estimate the clinical efficiency of immune checkpoint blockade (ICBs) therapy between two groups (**Table S11**). We accordingly found that the TIDE scores in the low TMEscore group were calculated significantly lower than that in the high TMEscore group (Wilcoxon test, *p* = 6.9e−06). The higher TIDE predictive scores in tumor correlated with worse effect of ICBs and poor survival outcomes. In the current analysis, we thus speculated that KIRC patients with higher TMEscores might benefit from better ICB efficiency and optimistic prognosis (**Figure 8B**). Additionally, we also conducted the receiver operating characteristic (ROC) curves to compare the differential power in predicting efficiency of ICBs between traditional TMB and established TMEscores, in which the TIDE scores were defined as the observation value. Overall, the TMEscore was proved to be an effective indicator for tumor prognosis and ICB efficiency based on Bootstrap test with *p*-value = 0.0436 (**Figure 8C**).

## Discussion

In this study, we integrated the multi-omics data of ccRCC cohorts (TCGA-KIRC, ICGC-ccRCC) to depict the genomic features of TME and evaluate the prognostic significance of TMEscore in clinical utility, especially guiding more valuable immunotherapy strategies. Firstly, we used the CIBERSORT to infer the fractions of immune-infiltrating cells in TME and exhibit the relatively specific profiles of immune cells. Subsequent consensus clustering analysis indicated that patients in TMEcluster5 showed better prognosis, in which the memory B cell accounted for the most abundant infiltrating proportions. Accordingly, the differential genes among the 5 clusters indicated that the functional enrichment pathways were humoral immune response and immunoglobulin-mediated immune response, especially the B-cell-mediated immunity. Meanwhile, we calculated the TMEscore as a robust prognostic factor, in which patients with a lower TMEscore suffer from worse survival outcomes. Mutational analysis revealed the differential mutation burden between two TME groups, especially the gene clusters of UBBP4, SPEN, BDP1, and DNAH2, and we further uncovered the associations of the low TMEscore group with signature6, signature5, and signature24 included in the COSMIC dataset (29).

Blockade drugs targeting the PD1/PDL1 signaling pathway have brought novel insights into the treatment strategies and exhibit relatively satisfactory efficiency across multiple tumors, including breast cancer, colon cancer, bladder cancer, advanced gastric cancer, and kidney cancer (30–32). However, the powerful biomarkers are warranted for predicting the drug response of immune checkpoint inhibitors. Previous researchers found that PD-1 or PD-L1 expression levels, MSI status, and mutational load were not ideal factors for predicting the benefits of drugs with lower sensitivity (33–35). So far, whether TMB correlates with improved survival outcomes or promotion of immunotherapies remains controversial in ccRCC and large cohorts are needed to validate its significance (36). As a result, it is urgent to develop a predictive biomarker of checkpoint immunotherapy for maximizing the therapeutic benefit. Intensive research has revealed the crucial role of TME in checkpoint inhibitor immunotherapy (37, 38). In our study, we further integrated the comprehensive landscape of interactions across tumor-infiltrating immune cells and relationships with clinical characteristics of renal cancer.

Our study suggested that the quantified TMEscore was a prognostic factor for ccRCC and significantly correlated with molecular sub-clusters. Dongqiang Zeng et al. have already uncovered the strong positive associations of TMEscore with mutation burden and predicted neo-antigen burden in more than 1,000 gastric tumor patients (32, 39). So far, the predictive biomarkers in clinical utility and strategies to assess clinical response have mainly shown interest in T-cell compartment, partially ignoring the contributions to anti-tumor immunity of other immune subsets (40, 41). However, Petitprez et al. confirmed the significance of immune subtypes in patients with soft-tissue sarcoma and disclosed the potential ability of B cells in forming tertiary lymphoid structures to guide new insights into clinical treatments (42). Of note, Helmink et al. further demonstrated and supported that the memory B cells were enriched in the tumors of responders for ICB treatment, implicating the foreground of novel biomarkers (43). Furthermore, our results suggested that a higher TMEscore was positively related to the subgroup of TMEcluster5, in which memory B cells revealed higher infiltrating levels compared with other immune cells. Meanwhile, patients with a higher TMEscore were associated with better ICB therapeutic efficiency, emphasizing that memory B-cell activation was the core mechanism of sensitivity to checkpoint blockade. These findings might facilitate the precise immunotherapy strategy and the combined management of both activation of memory B cell and traditional ICB. In addition, Netti et al. also found that PTX3 could modulate the immunoflogosis in TME and function as a prognostic factor for patients with ccRCC (44). Analysis of complement system activation on tumor tissues showed the co-expression of PTX3 with C1q, C3aR, C5R1, and CD59. The expression of PTX3 can affect the immunoflogosis in the ccRCC microenvironment, by activating the classical pathway of CS (C1q) and releasing pro-angiogenic factors (C3a and C5a). The upregulation of CD59 also inhibits the complement-mediated cellular lysis. Although this study uncovered the tight associations between PTX3 and TME remodeling, the predictive efficiency of PTX3 in ccRCC was not well elucidated and validated. Our established signature is a model constructed by multiple genes and has strong robustness. Calculation and application of our TMEscore would be useful for clinical prediction and ICB treatment of ccRCC.

Apart from the associations with immune filtration analysis, we further discussed the TMEscore with mutation profiles in ccRCC. We completely illustrated the mutational landscape in both groups and found the differential mutated frequencies of drivers between two TMEscore levels, including UBBP4, SPEN, and BDP1. In particular, Sharma et al. (45) also reported that the deleterious mutation of SPEN p.S1078* emerges as a putative potential therapeutic target in advanced-stage urothelial carcinoma. Additionally, we first analyzed the relationships between mutated distributions with a specific signature in the COSMIC dataset in two TMEscore groups. The included signature6 and signature24 were significantly associated with defective DNA mismatch repair, chromosomal instability, and exposure of aflatoxin, which might be one of the underlying explanations for the patients with poor survival outcomes in the low TMEscore group. Furthermore, we still detected the associations of mutation features in high TMEscore with signature5 and signature12 matched with unknown etiology annotations, and large cohorts with in-depth somatic mutated sequencing are warranted. Of note, CNV data were utilized to infer the tumor purity and ploidy and there was no significant difference between two TMEscore groups. We thus speculated that tumor purity might not function as a vital determinant for prognosis in two TMEscore levels. Moreover, differential analysis revealed the significant immune-related miRNAs (hsa-miR-29b-3p, hsa-miR-139-5p, and hsa-miR-142-5p), among which Montero-Conde et al. supported the idea that the hsa-miR-139-5p/HNRNPF axis served as a novel regulatory mechanism associated with the modulation of major thyroid cancer signaling pathways and tumor virulence (46, 47). Subsequent Kaplan–Meier analysis further identified 19 TMEscore-related prognostic miRNAs and 23 hub mRNAs (SAA1, SLC27A2, PPP1R1A, SAA2, etc.). We last drew the heatmap to exhibit the top 23 TMEscore-related hazard signatures combined with integrative analysis of TMB, purity, and subclusters between two groups, providing significantly therapeutic targets for ccRCC.

However, there were still several limitations in our work. Firstly, the fractions or prognostic value of TMEscore-related immune cells or risk signature are not validated by flow cytometry or other experimental studies. Apart from only two centers of TCGA or ICGC patients, other independent cohorts in our hospitals with more ccRCC patients were warranted to further support the clinical significance of TMEscore in predicting prognosis. Furthermore, we just utilized the TIDE dataset to estimate the ICB efficiency and we should enroll eligible patients treated with ICB drugs to compare the indicative value of TMEscore with TMB. As we observed that not all patients with high TMEscore gained better benefit of immunotherapy, more clinical factors should be incorporated to integrative models for the improvement of predictive accuracy.

Overall, our study systematically revealed unique biological insights into TMEscore in ccRCC, the major subtype of kidney cancer, combining complementary genomic, transcriptome, and mutation profiles with clinical characteristics, providing an invaluable bioinformatic resource for subsequent research on TME in ccRCC tumorigenesis. For the first time, we characterize in detail the TME subcluster, the establishment of TMEscore, the potential associations of TMEscore with mutation profiles, CNV burden, risk signature, and ICB efficiency. The TMEscore, as we have quantified, was proved to have promising significance for predicting prognosis and ICB responses, in accordance with the goal of developing rationally individualized therapeutic interventions.

## Data Availability Statement

The original contributions presented in the study are included in the article/**Supplementary Material**. Further inquiries can be directed to the corresponding authors.

## Author Contributions

CZ: formal analysis, investigation, visualization, writing—original draft, and writing—review and editing. FQ: formal analysis, data curation, methodology, software, and writing—review and editing. YZ: formal analysis, data curation, methodology, supervision, validation, and writing—review and editing. XX: data curation. XL and XW: conceptualization, funding acquisition, project administration, resources, supervision, and writing—review and editing. All authors contributed to the article and approved the submitted version.

## Funding

This work was supported by grants from the Medical Research Project of Jiangsu Provincial Health and Family Planning Commission (No. H2018052), and the Young Talents Program of Jiangsu Cancer Hospital (No. 2017YQL-04).

## Conflict of Interest

The authors declare that the research was conducted in the absence of any commercial or financial relationships that could be construed as a potential conflict of interest.

## Publisher’s Note

All claims expressed in this article are solely those of the authors and do not necessarily represent those of their affiliated organizations, or those of the publisher, the editors and the reviewers. Any product that may be evaluated in this article, or claim that may be made by its manufacturer, is not guaranteed or endorsed by the publisher.
