# Supplementary figures and images for: Comprehensive Genomic Characterization of Tumor Microenvironment and Relevant Signature in Clear Cell Renal Cell Carcinoma
Source: Front Oncol. 2022 May 16;12:749119. doi: 10.3389/fonc.2022.749119 (PMC9149313; doi:10.3389/fonc.2022.749119)

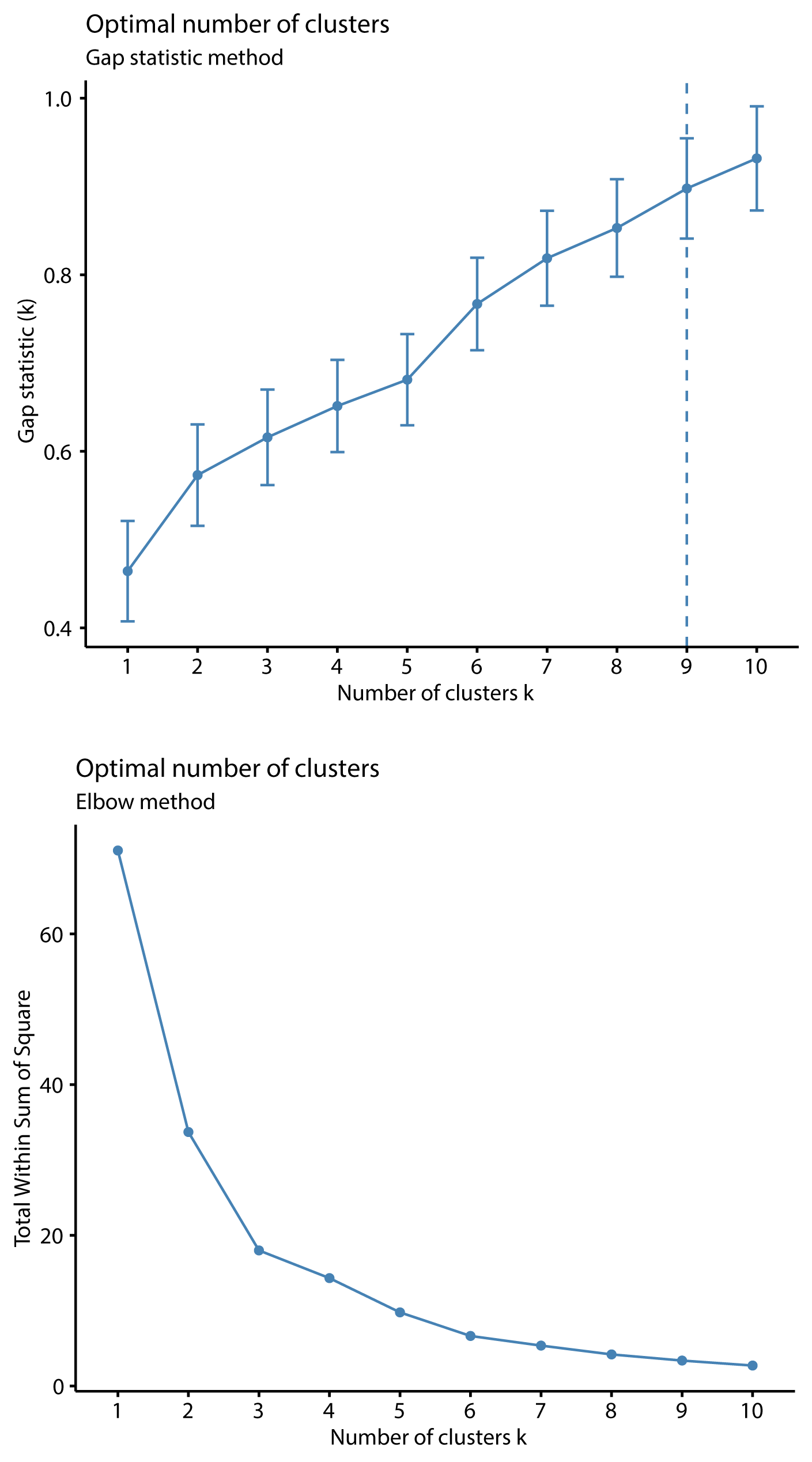

Supplement: Supplementary file 1 [file Image_1.tif]

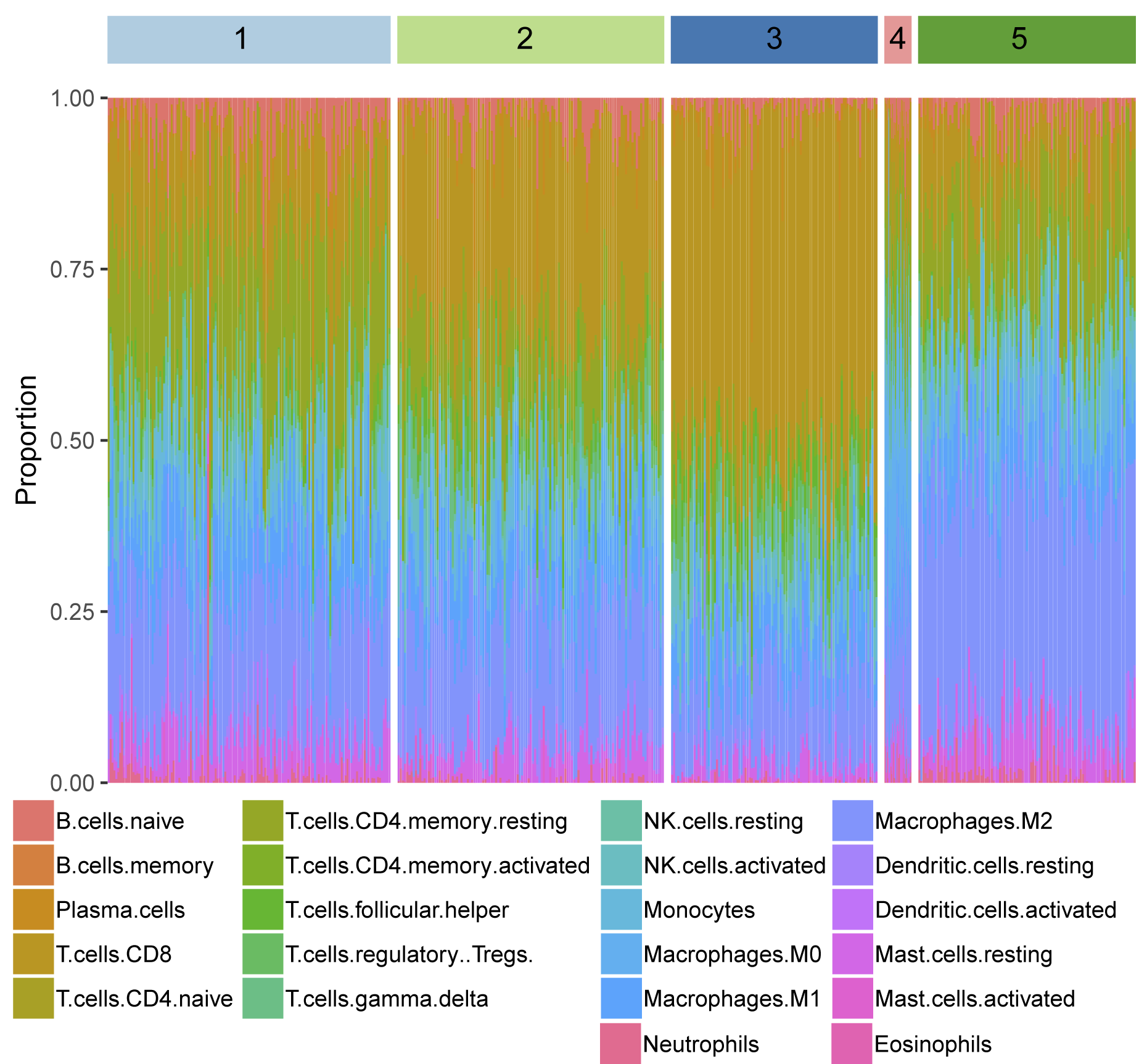

Supplement: Supplementary file 2 [file Image_2.tif]

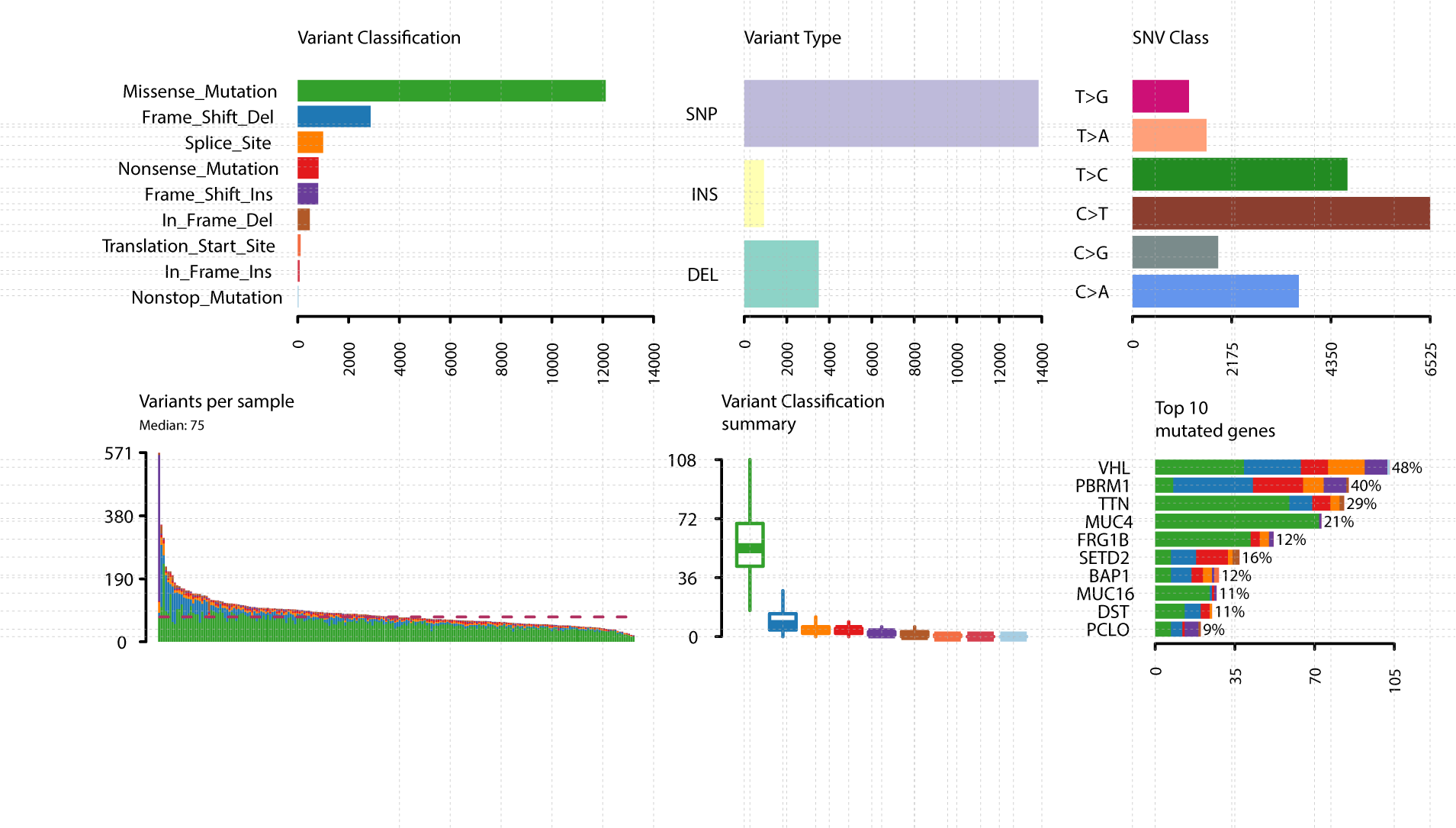

Supplement: Supplementary file 3 [file Image_3.tif]

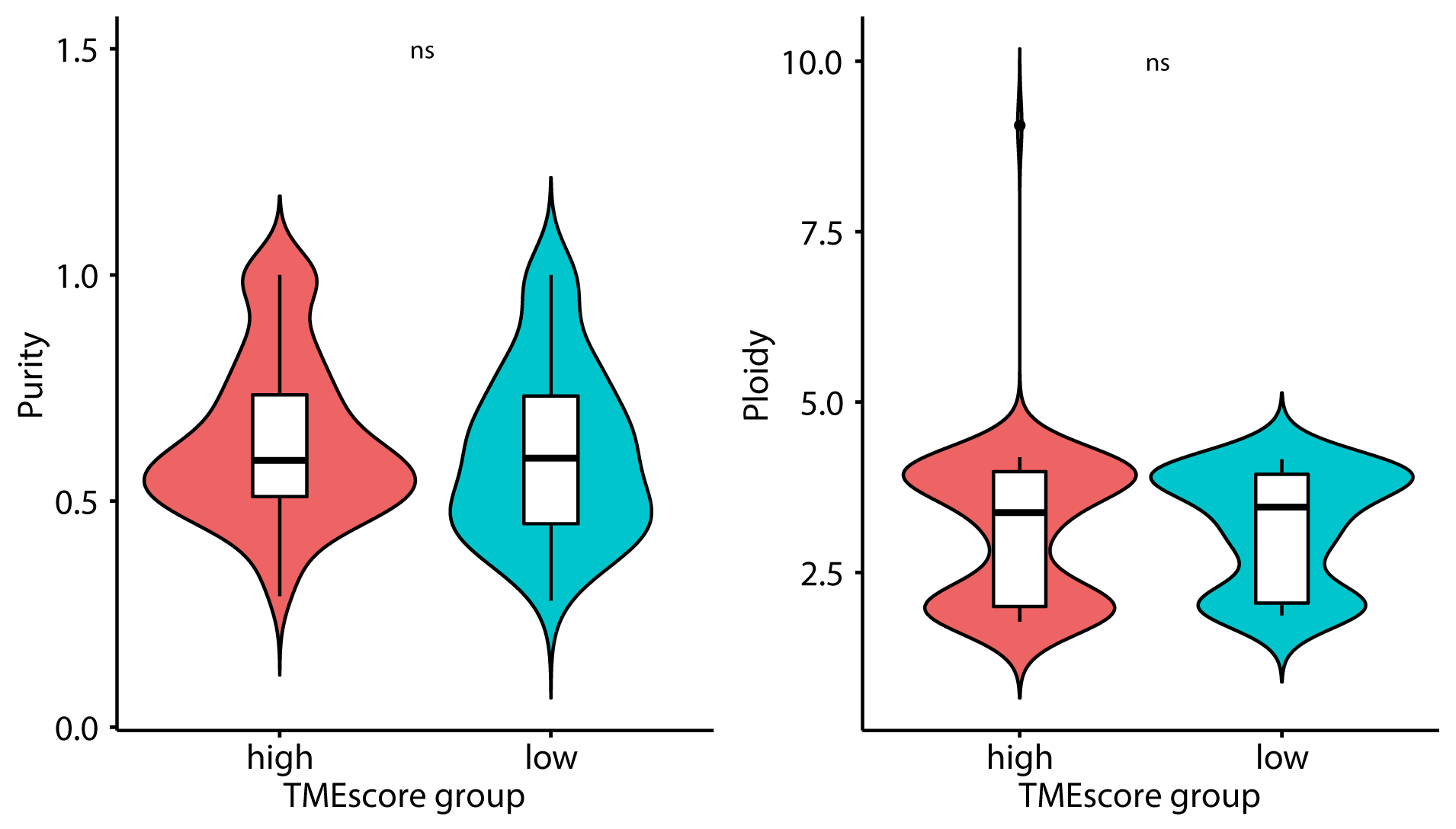

Supplement: Supplementary file 4 [file Image_4.tif]

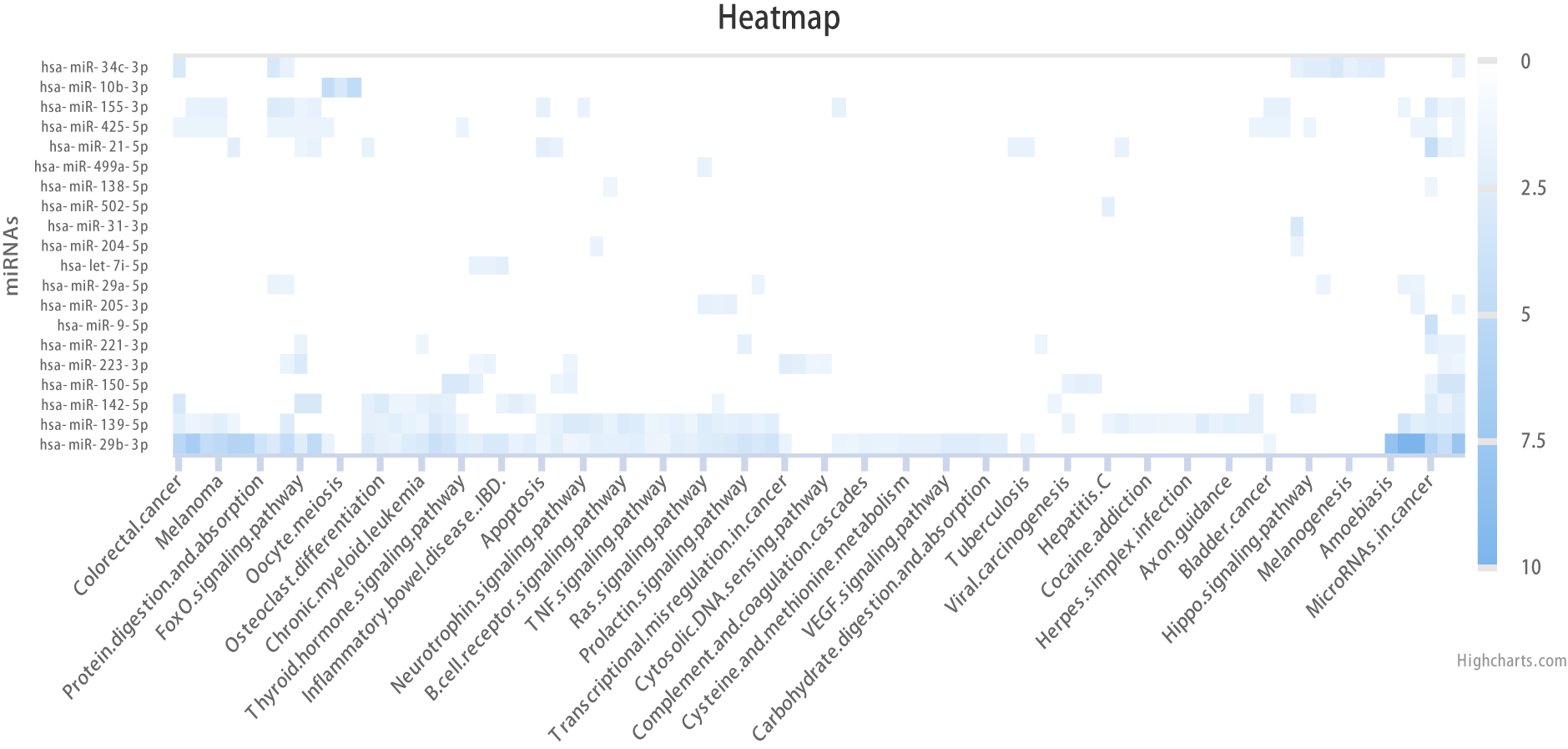

Supplement: Supplementary file 5 [file Image_5.tif]

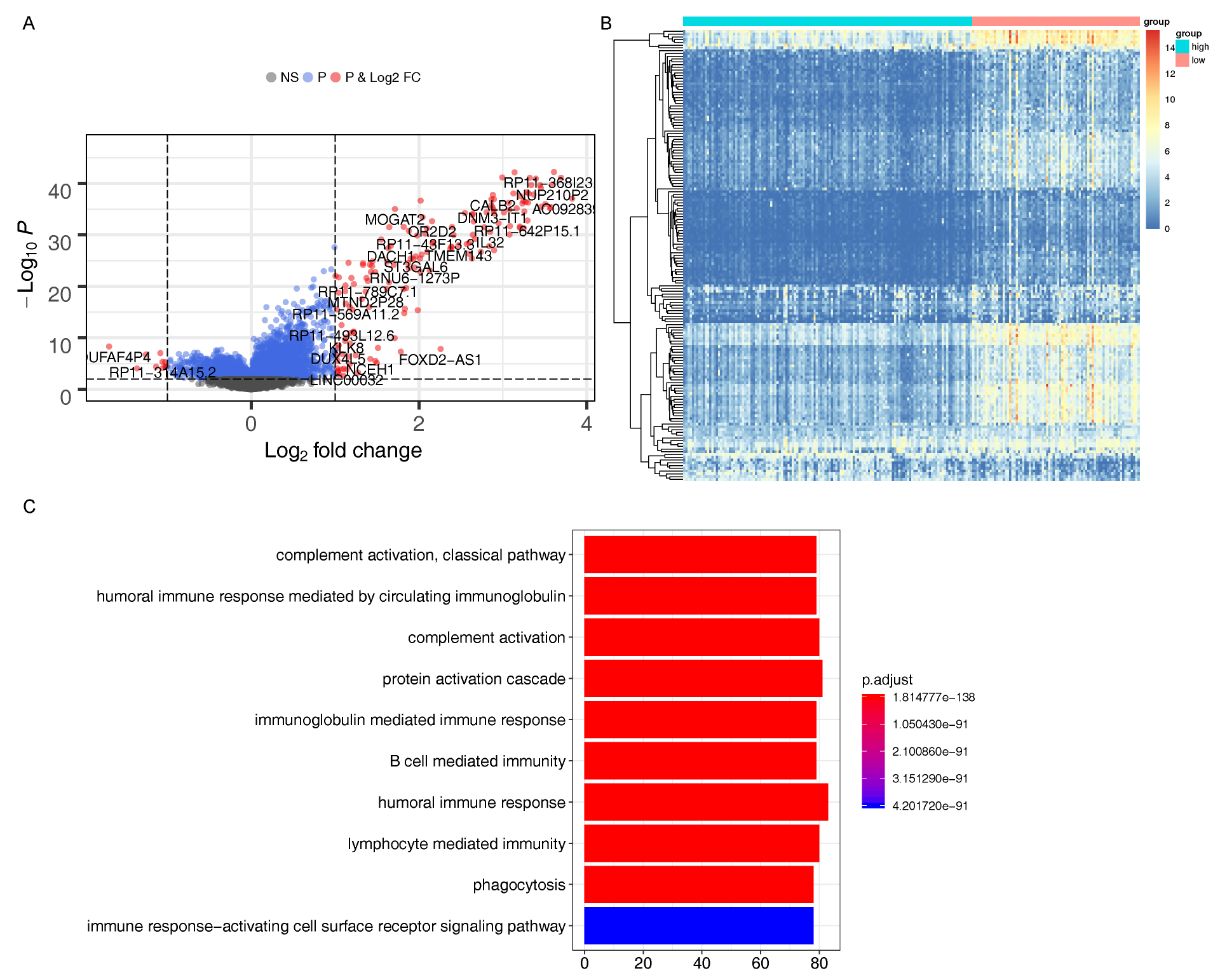

Supplement: Supplementary file 6 [file Image_6.tif]
